# Deletion of African Swine Fever Virus Histone-like Protein, A104R from the Georgia Isolate Drastically Reduces Virus Virulence in Domestic Pigs

**DOI:** 10.3390/v14051112

**Published:** 2022-05-22

**Authors:** Elizabeth Ramirez-Medina, Elizabeth A. Vuono, Sarah Pruitt, Ayushi Rai, Nallely Espinoza, Alyssa Valladares, Ediane Silva, Lauro Velazquez-Salinas, Manuel V. Borca, Douglas P. Gladue

**Affiliations:** 1 Plum Island Animal Disease Center, Agricultural Research Service, United States Department of Agriculture Greenport, Greenport, NY 11944, USA; elizabeth.ramirez@usda.gov (E.R.-M.); elizabeth.vuono@usda.gov (E.A.V.); sarah.pruitt@usda.gov (S.P.); ayushi.rai@usda.gov (A.R.); nallely.espinoza@usda.gov (N.E.); alyssa.valladares@usda.gov (A.V.); ediane.silva@usda.gov (E.S.); lauro.velazquez@usda.gov (L.V.-S.); 2 Department of Pathobiology and Population Medicine, Mississippi State University, P.O. Box 6100, Starkville, MS 39762, USA; 3 Oak Ridge Institute for Science and Education (ORISE), Oak Ridge, TN 37830, USA; 4 College of Veterinary Medicine, Kansas State University, Manhattan, KS 66506, USA

**Keywords:** ASFV, ASF, African swine fever virus, virus neutralizing antibodies, protective immunity

## Abstract

African swine fever virus (ASFV) is the etiological agent of a frequently lethal disease, ASF, affecting domestic and wild swine. Currently, ASF is causing a pandemic affecting pig production in Eurasia. There are no vaccines available, and therefore control of the disease is based on culling infected animals. We report here that deletion of the ASFV gene A104R, a virus histone-like protein, from the genome of the highly virulent ASFV-Georgia2010 (ASFV-G) strain induces a clear decrease in virus virulence when experimentally inoculated in domestic swine. A recombinant virus lacking the A104R gene, ASFV-G-∆A104R, was developed to assess the role of the A104R gene in disease production in swine. Domestic pigs were intramuscularly inoculated with 10^2^ HAD_50_ of ASFV-G-∆A104R, and compared with animals that received a similar dose of virulent ASFV-G. While all ASFV-G inoculated animals developed a fatal form of the disease, animals receiving ASFV-G-∆A104R survived the challenge, remaining healthy during the 28-day observational period, with the exception of only one showing a protracted but fatal form of the disease. ASFV-G-∆A104R surviving animals presented protracted viremias with reduced virus titers when compared with those found in animals inoculated with ASFV-G, and all of them developed a strong virus-specific antibody response. This is the first report demonstrating that the A104R gene is involved in ASFV virulence in domestic swine, suggesting that A104R deletion may be used to increase the safety profile of currently experimental vaccines.

## 1. Introduction

African swine fever virus (ASFV) is the causative agent of African swine fever (ASF), a frequently lethal disease affecting domestic pigs and currently damaging the swine production industry in Eurasia. The disease has just been discovered in the Dominican Republic and Haiti after more than 40 years of being absent in the Western hemisphere [1]. No commercial vaccines are available; therefore, disease control is restricted to culling all infected animals and limiting the mobilization of infected animals.

ASFV is a very large and structurally complex virus, with a large (180–190 kilobase pairs) double-stranded DNA genome encoding more than 150 genes [2]. Due to a large number of genes in ASFV, most of these genes are understudied. The molecular function of many of these genes remains undefined, with, at best, a predicted function based on sequence similarities to other virus or mammalian genes. Recent studies deleting single genes in the ASFV Georgia strain have resulted in varying phenotypes from undetectable or minor changes to virus growth in primary swine macrophages or in virulence (i.e., A859L [3], KP117R [4], MGF110-1L [5], X69R [6], MGF360-1L [7], and CD2 [8]).

Effective experimental vaccines are usually based on the use of attenuated strains of viruses, particularly those developed by the deletion of specific virus genes involved in disease production from the genome of virulent field ASFV isolates. Therefore, the identification of genes involved in virus virulence in pigs is an essential initial step in the rational design of live attenuated ASFV vaccine candidates. Using this approach, several experimental recombinant vaccine candidates have been developed which efficiently protect pigs against the challenge of the ASFV-G isolate or its field isolate derivatives [9,10,11]. Therefore, the discovery of virus genes important in the process of disease production is a critical first step in the design of recombinant live attenuated vaccines (LAVs) to prevent ASFV infection and disease.

The discovery of novel determinants of virulence is important for several reasons. The inclusion of additional gene deletions could increase the safety profile of some preexisting vaccine candidates or single-gene deletions with residual virulence [12,13]. In addition, it has been repeatedly observed that the deletion of even highly conserved virus genes may have different effects on the virulence of the resulting virus, depending on the virus strain considered [12,14,15,16]. Therefore, the discovery and characterization of new genetic determinants of virulence are critical for the rational development of the next generation of live attenuated vaccine candidates, whether to increase the safety profile of a current potential vaccine strain that has residual virulence or to achieve the attenuation in novel emerging ASFV isolates.

Here, we identified a novel determinant of virulence, the ASFV gene A104R. The protein encoded by the A104R gene has been shown to function as a histone-like protein, closely associated with virus DNA in the virus particle [17]. In addition, the A104R encoded protein (pA104R) was shown to bind double-strain DNA, and even its critical residues mediating the function have been identified [18]. In addition, siRNA experiments performed using an ASFV adapted to grow in Vero cells demonstrated that pA104R plays a critical role in virus replication [18]. Nevertheless, there is no information regarding pA104R function in virus replication in the natural target cell, swine macrophages, and in the process of virus virulence in domestic swine. We demonstrated here that a recombinant virus harboring a deletion of the A104R from the genome of virulent ASFV-G isolate (ASFV-G-∆A104R) has a decreased ability to replicate in swine macrophages cultures and has a drastic reduction in its virulence when inoculated in swine when compared with the virulent parental ASFV-G.

## 2. Materials and Methods

### 2.1. Viruses and Cells

Primary cultures of blood-derived swine macrophages were performed as previously described [19] and seeded at a density of 5 × 10^6^ cells per ml. ASFV Georgia (ASFV-G) was a field isolate kindly provided by Dr. Nino Vepkhvadze, from the Laboratory of the Ministry of Agriculture (LMA) in Tbilisi, Republic of Georgia [20]. Growth curves between ASFV-G-∆A104R and parental ASFV-G were performed in primary swine macrophage cell cultures at an MOI of 0.01 HAD_50_ (hemadsorbing doses, as determined in primary swine macrophage cell cultures) as previously described [4].

### 2.2. Virus Titrations

Sample points were taken at 2, 24, 48, 72, and 96 h, cells were frozen at ≤−70 °C, thawed, and the lysates titrated by HAD_50_/mL in primary swine macrophage cell cultures in 96-well plates. The presence of the virus-infected cells was assessed by hemadsorption (HA) and virus titers were calculated as previously described [21].

### 2.3. Construction of the ASFV A104R Deletion Mutant

A virus with a deleted A104R gene (ASFV-G-∆A104R) was obtained by homologous recombination between the genome of the parental ASFV-G and a recombination transfer vector as previously described [22]. The recombinant transfer vector (p72mCherryΔA104R) contains both flanking genomic regions of the A104R gene: the left arm spans between genomic positions 47,331–48,331 while the right arm is situated between genomic positions 48,608–49,608, and contains a reporter gene cassette containing the mCherry fluorescent protein (mCherry) gene under the control of the ASFV p72 late gene promoter [23]. The recombinant transfer vector was obtained by DNA synthesis (Epoch Life Sciences, Sugar Land, TX, USA). As designed, this construction created a 276-nucleotide deletion between nucleotide positions 48,332–48,607, deleting the A104R ORF sequence mutant ASFV-G-∆A104R was purified by consecutive limiting dilution steps based on mCherry activity detection. ASFV-G-∆A104R stock was full-length sequenced using next-generation sequencing (NGS).

### 2.4. Next-Generation Sequencing of ASFV

ASFV DNA was extracted from infected cell cultures showing 90–100% CPE, using the nuclear extraction kit (Active Motif cat# 40010). The nucleus and cytoplasmic fractions were separated, and the cytoplasmic fraction was used to isolate the viral DNA, following the manufacturer’s protocol. In brief, ASFV infected cells were collected and incubated in the hypotonic buffer for 15 min on ice or until the cell membrane was dissolved. The nucleus fraction was separated by centrifugation. The cytoplasmic fraction was collected and DNA was extracted by adding 10% 3 M NaOAc by volume to the sample (Sigma-Aldrich 71196) and an equal volume of phenol:chloroform:isoamyl alcohol (25:24:1) with a pH of 6.5–6.9 (Sigma-Aldrich P3803-100ML, St. Louis, MO, USA), and centrifuged for max speed in a tabletop centrifuge. The aqueous layer was then ethanol precipitated using 2 volumes of 100% ethanol, washed with the same volume of 70% ethanol, and dried. The resulting DNA pellet is then reconstituted in sterile water. We then used this DNA library for NGS sequencing using Nextera XT kit in the NextSeq (Illumnia, San Diego, CA, USA) following the manufacturer’s protocol. Sequence analysis was performed using CLC Genomics Workbench software (CLCBio, Waltham, MA, USA) 2.3.

### 2.5. Evaluation of ASFV-G-ΔA104R Virulence in Domestic Pigs

Virulence of ASFV A104R deletion mutant was assessed in 35–40 kg commercial breed swine. Five pigs were intramuscularly (IM) inoculated with 10^2^ HAD_50_ of ASFV-G-∆A104R and compared with a group of five pigs inoculated with 10^2^ HAD_50_ of ASFV-G. Clinical signs (anorexia, depression, fever, purple skin discoloration, staggering gait, diarrhea, and cough) and changes in body temperature were recorded daily throughout the experiment. Blood samples were scheduled to be obtained at 0, 4, 7, 11, 14, 21, and 28 days post-inoculation (pi). Animal experiments were performed under biosafety level 3 conditions in the animal facilities at Plum Island Animal Disease Center, following a strict protocol approved by the Institutional Animal Care and Use Committee (225.06-19-R_090716, approved on 9 June 2019).

## 3. Results and Discussion

### 3.1. A104R Gene Is Conserved across Different ASFV Isolates

To predict the global genetic variability of A104R gene among ASFV strains from the field, we used the Georgia 2007/1 strain (GenBank access NC_044959.2) as a query to conduct a Blast analysis to obtain all A104R gene sequences reported on GenBank. A total of 100 sequences were downloaded and aligned by ClustalW, with redundancy removed using the software Jalview version 2.11.1.7. Final ASFV strains used to conduct the analyses reported here included Georgia 2007/1 (genotype II, and representative of the Eurasian lineage), K49 (genotype I), LO2018 (genotype I), Pretoriuskop/96/4 (genotype XX), Liv13/33 (genotype I), RSA 2 2004 (genotype XX), Mkuzi 1979 (genotype VII), SPEC 57 (genotype III), Warthog (genotype IV), Warmbaths (genotype III), and Tengani 62 (genotype V).

**Figure 1:**
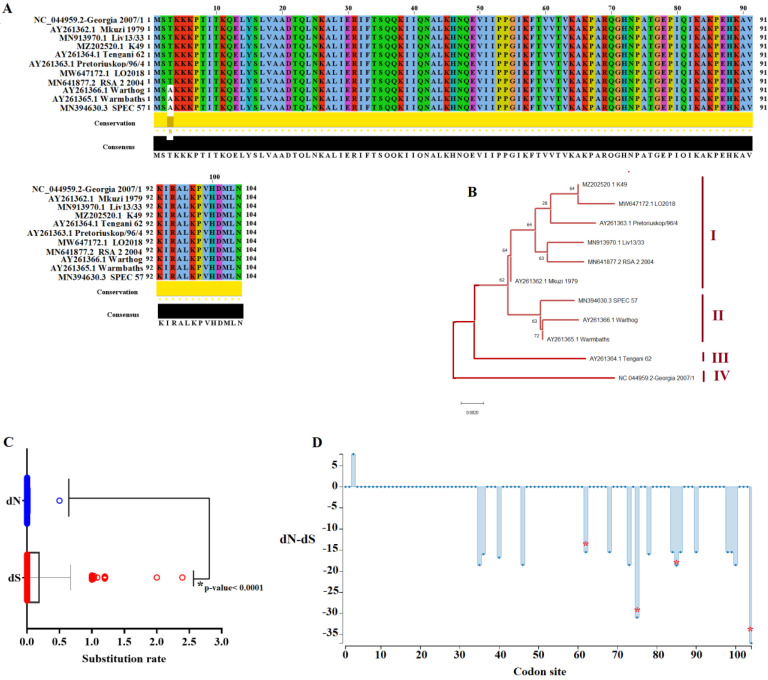
Evaluation of the A104R protein across ASFV isolates. (**A**) Amino acid alignment representing the diversity of A104R protein of ASFV in the field. Residues in white spots represent changes between amino acids with different charge. Conservation plot scores reflect the nature of the change in specific sites, with high scores associated with changes with similar biological properties. Alignment was produced using the software Jalview version 2.11.1.7. (**B**) Phylogenetic analysis conducted by maximum likelihood method and the Tamura-3 parameter model showing the diversity of A104R gene of ASFV in the field. Based on the cluster distribution, isolates were categorized in four main groups. Numbers above internal branches represent bootstrap values (1000 repetitions). (**C**) Comparison between the evolutionary rate of synonymous (dS) and nonsynonymous (dN) mutations during the evolution of A104R gene of ASFV. Significant differences between dS and dN rates were calculated by the unpaired *T* test. (**D**) The graphic represents the ratio dN-dS at specific codon sites in the A104R gene of ASFV. Red asterisks represent codon sites evolving under purifying selection. Analysis was conducted using the evolutionary algorithms FEL considering a cutoff value of *p* = 0.1.

Pairwise analysis conducted using the p-distance model and the bootstrap method as an approach to give statistical confidence (*p*-0.05) [24] revealed a between 97.11% and 99.67% (~98.59%) and between 100% and 99.03 (99.57%) at nucleotide and amino acid levels respectively, indicating the high conservation of A104R among ASFV isolates in the field. These results were consistent with previous analyses published during the 90’s [17,25], confirming the high conservation of this gene during the evolution of ASFV. In fact, the high conservation of this protein can be graphically visualized in Figure 1A, where a single amino acid substitution was observed at residue number three (T-A) of its 104 amino acid protein. This substitution was observed in isolates SPEC 57, Warthog, and Warmbaths. In the case of the Eurasian pandemic lineage, no nucleotide changes were observed in this gene among multiple isolates reported from the pandemic Eurasian lineage ranging from 2007 to 2020, indicating the high conservation of this gene during this pandemic.

However, despite the single amino acid substitution observed in A104R protein, when the phylogenetic analysis was conducted using nucleotide sequences of A104R, it was possible to predict the existence of four different phylogenetic groups (Figure 1B). The divergence between groups was highly supported by the accumulation of synonymous mutations. In this context, to assess the role of synonymous mutations during the evolution of A104R gene, we used the single-likelihood ancestor counting (SLAC) [26] evolutionary algorithm to predict the evolutionary rates of synonymous (dS) and nonsynonymous (dN). The results showed the preponderance of synonymous mutations during the evolution of the A104R gene, indicating that synonymous mutations accumulate 41 times faster than nonsynonymous mutations in these genes (Figure 1C). Furthermore, in the phylogenetic tree, the basal branch position of the Georgia 2007/1 isolates in relation with the rest of the isolates suggests that this gene may be use as a phylogenetic marker for molecular epidemiology analysis to determine the presence of the Eurasian pandemic lineage. Interestingly, we found different putative single nucleotide polymorphisms (SNPs) in the pandemic lineage, all of them accounting for synonymous substitutions at codons 40 (ATA-ATC), 62 (ACG-ACA), 73 (AAT- AAC), 75 (GCC-GCA), and 84 (GCT-GCC).

Finally, to attain more insights into the evolution of A104R gene, we conducted different evolutionary tests on the evolutionary server Datamonkey [27]. No evidence of recombination events was predicted in the A104R gene using the genetic algorithm for recombination detection (GARD) [28]. Overall dN/dS values = 0. 024 were predicted by the algorithm SLAC, indicating that purifying selection is the main force driving the evolution of this gene. This result is consistent with the high levels of identity observed among ASFV isolates, indicating the essential function of A104R in ASFV. In this sense, previous studies have determined the essential roles of A104R is a histone-like protein, participating during the process of viral replication, transcription, and packing [18,29], and thus explaining the high conservation of A104R protein during the evolution of ASFV.

Using the algorithm fixed effects likelihood (FEL) [26], a total of 4 codons were found experimenting with the purifying selection, indicating the potential relevance of residues 62, 75, 85, and 104 in the functionality of this protein (Figure 1D). In this context, residue 85 (K) is associated with DNA bringing interactions [29]. No evidence of positive selection was found in the unique amino acid change observed in this protein when assessed by FEL or mixed-effects model of evolution (MEME) [30] algorithms, suggesting that change at position 3 (T-A) may represent a neutral change.

### 3.2. Development of the ASFV-G-ΔA104R Deletion Mutant

The high level of nucleotide and amino acid conservation of the A108R gene among different ASFV isolates and along with its function as a DNA binding protein [31] suggest that A104R may play an important role in the process of virus replication.

**Figure 2:**
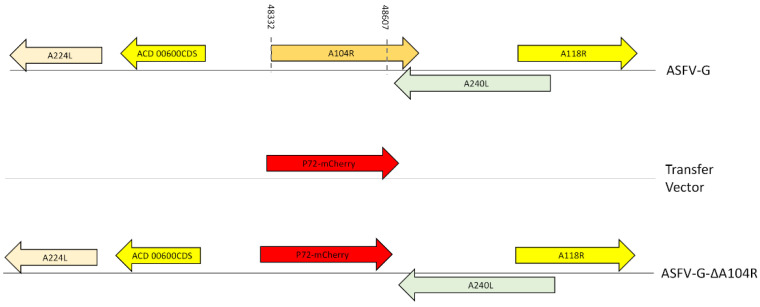
Schematic for the development of ASFV-G-∆A104R. The transfer vector contains the p72 promoter and an mCherry cassette; the gene positions are indicated. The homologous arms were designed to have flanking ends to both sides of the deletion/insertion cassette. The nucleotide positions of the area that was deleted in the ASFV-G genome are indicated by the dashed lines. The resulting ASFV-G-∆A104RL virus with the cassette inserted is shown on the bottom.

To assess the potential critical role of A104R during the process of virus replication in cell swine macrophages and in infected animals, a recombinant virus with the A104R gene deleted was developed (ASFV-G-∆A104R) using the highly virulent ASFV Georgia 2007 isolate (ASFV-G) as the parental virus. The A104R gene was deleted by replacing 92 amino acid residues of the A104R ORF with the p72mCherry cassette by homologous recombination [28]. An area covering 276 bp (between nucleotide positions 48,332 and 48,607) was eliminated from the genome of ASFV-G, deleting the majority of the A104R gene and leaving only the last 29 bp of the C-terminus so as not to disturb the C-terminus of A240L which overlaps. The C-terminus was then substituted with a 1226-bp cassette containing the p72mCherry construct (see Material and Methods) (Figure 2). The recombinant ASFV-G-∆A104R stock was purified after successive limiting dilution steps using primary swine macrophage cell cultures.

To evaluate the accuracy of the alterations induced into the ASFV-G-∆A104R genome, the full genome sequence was obtained by NGS using an Illumina NextSeq^®^ 500. A total of 3,189,814 reads were aligned to the ASFV genome. A comparative study between genomes of ASFV-G-∆A104R and ASFV-G demonstrates a deletion of 276 nucleotides, and an insertion of 1226 nucleotides corresponding to the insertion of the p72-mCherry cassette sequence. No undesired additional changes were detected as result of the process of production and purification of ASFV-G-∆A104R. In addition, NGS data showed the absence of parental ASFV-G genome as a potential contaminant in the ASFV-G-∆A104 stock.

### 3.3. Replication of ASFV-G-∆A104R in Primary Swine Macrophages

**Figure 3:**
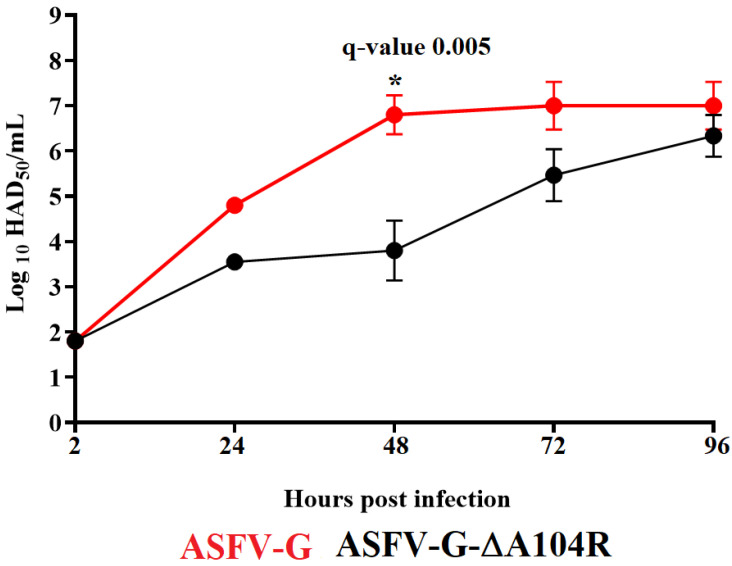
In vitro growth kinetics in primary swine macrophage cell cultures for ASFV-G-∆A104R and parental ASFV-G (MOI= 0.01). Samples were taken from three independent experiments at the indicated time points and titrated. Data represent means and standard deviations of three replicas. Sensitivity using this methodology for detecting virus is ≥log10 1.8 HAD_50_/mL. (*) Indicates significant differences.

To analyze the potential role of the A104R gene during the process of virus replication, the ability of ASFV-G-∆A104 to replicate in swine macrophages was evaluated and compared to that of the parental ASFV-G in a multistep growth curve in primary swine macrophage cultures. Macrophage cultures were infected at an MOI of 0.01 with either ASFV-G-∆A104R or the parental ASFV-G. Virus yields were assessed at 2, 24, 48, 72, and 96 h post-infection (pi). Results demonstrated that ASFV-G-∆A104R showed a reduction in virus yields of almost 1000 times at 48 h pi time point, although no significant differences were detected in virus yields at the end of the experiment (90 h pi) (Figure 3). Therefore, although deletion of the A104R gene from the ASFV-G genome does not affect final virus yields, it did produce a delayed replication kinetics. This is an interesting result, considering previous results showed that inhibition of A104R expression by siRNA severely affects virus replication (over 80%) with a reduced transcription of late viral genes [18]. In addition, attempts to develop and purify a recombinant virus lacking the A104R gene were unsuccessful, even by using a susceptible helper cell line expressing the A104R gene [32]. The referred results were obtained using ASFV BA71V, a virus strain adapted to grow in the Vero cell. Perhaps differences between those results and ours reside in the different virus model used to perform the studies. While we used an ASFV field isolate, ASFV-G, and the natural target cell to produce and test our recombinant virus ASFV-G-∆A104R, the referenced reports present results produced by using a virus, ASFV BA71V that has been adapted to growth in a monkey derived cell line, Vero. Adaptation to growth in Vero cell causes a large deletion on the left variable region of ASFV BA71V strain, causing the loss of 8–10 genes of the MGF360/505 [20]. Perhaps those genomic differences are responsible for the differential effect of the deletion of the A104R gene in ASFV-G and Ba71V.

### 3.4. Assessment of ASFV-G-∆A104R Virulence in Swine

**Figure 4:**
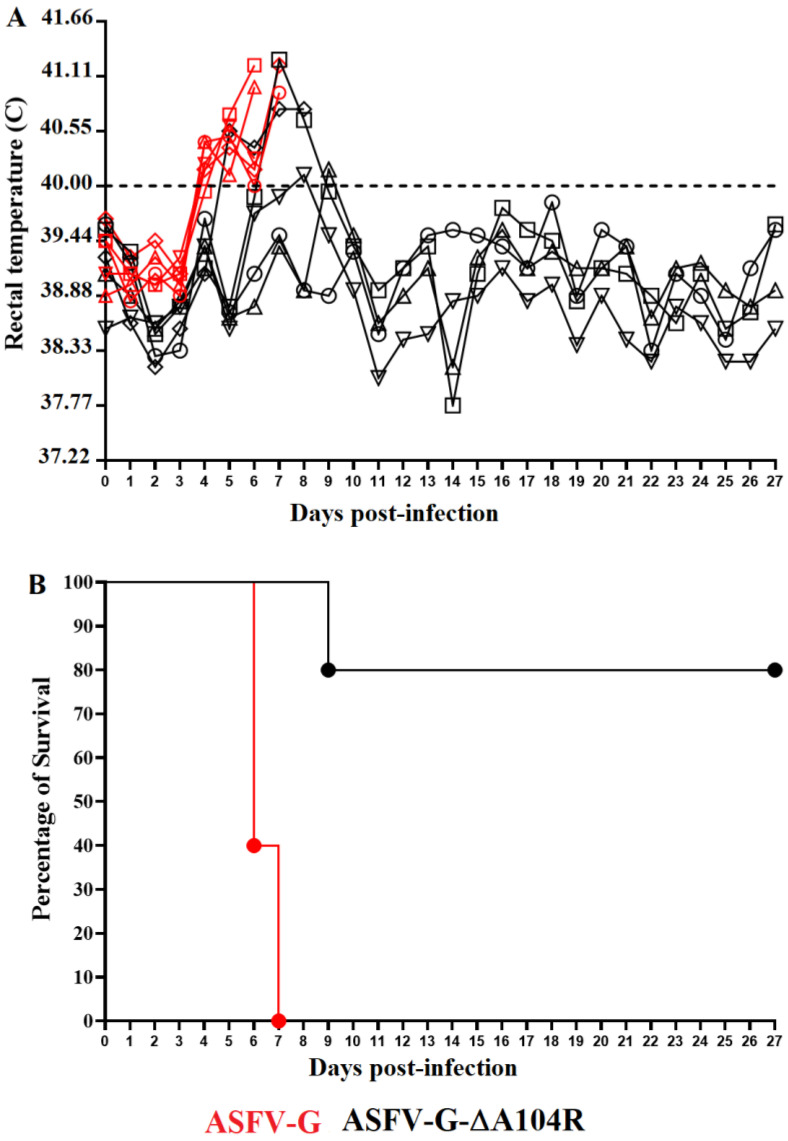
Evolution of body temperature (**A**) and mortality (**B**) in animals (5 animals/group) IM infected with 10^2^ HAD_50_ of either ASFV-G-∆A104R or parental ASFV-G.

To evaluate the effect of deleting the A104R gene from the genome of ASFV-G in virus virulence, ASFV-G-∆A104R was inoculated in a group of five domestic pigs by the IM route at a dose of 10^2^ HAD_50_. The clinical evolution of that group was monitored for 28 days and compared with that of a control group, which was also IM inoculated, but with 10^2^ HAD_50_ of ASFV-G. All animals inoculated with the parental virulent ASFV-G presented an increase in body temperature (>40 °C F) on 4–5 day pi which quickly evolved by the development of full clinical disease (depression, anorexia, staggering gait, diarrhea, and purple skin discoloration) (Table 1 and Figure 4) with all animals euthanized by day 6–7 pi.

Animals inoculated with ASFV-G-∆A104R survived the infection with the exception of one animal that developed a fatal form of the disease, similar to the one presented in the ASFV-G inoculated animals. This animal had a rise in body temperature by day 5 pi and was euthanized on day 8 pi. Three of the remaining animals presented just a transient rise of body temperature by days 7–9 pi, not accompanied by any clinical sign of ASF. The fifth animal remained free of any clinical signs related to the disease during the 28-day observational period. These results indicate that deletion of the A104R gene from the genome of ASFV-G provokes a clear decrease in virus virulence in experimentally infected domestic swine.

**Figure 5:**
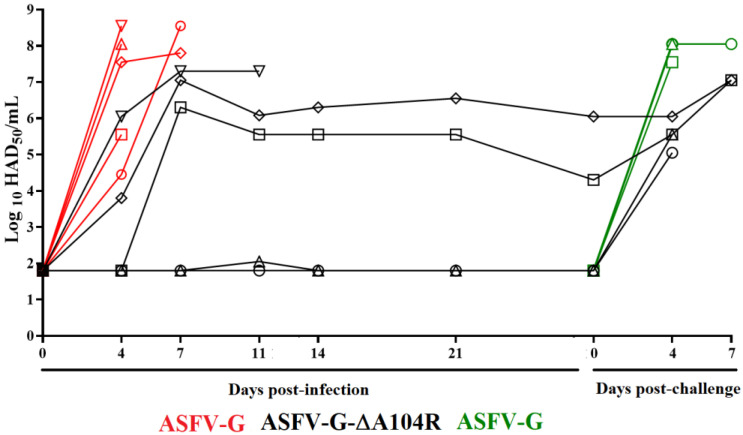
Viremia titers detected in pigs IM inoculated with 10^2^ HAD_50_ of either ASFV-G-∆A104R (filled symbols), or ASFV-G (empty symbols). Each symbol represents the average of animal titers in each of the groups. Sensitivity of virus detection: >log10 1.8 TCID_50_/mL.

Replication in the animals of either ASFV-G-∆A104R or ASFV-G was evaluated by measuring viremia titers throughout the 28-day experimental period (Figure 5). As expected, viremias in animals inoculated with parental ASFV-G had high titers (ranging from 10^4.55^–10^8.55^ HAD_50_/mL) at day 4 pi, remaining high until the day all animals were euthanized. Viremias in animals inoculated with ASFV-G-∆A104R presented a heterogeneous pattern. The animal which developed a lethal disease showed high virus titers (10^6^ HAD_50_/mL) by day 4 pi, with increasingly higher titers (10^7.33^ HAD_50_/mL) until it needed to be euthanized due to the severity of the clinical disease. Two of the other animals that survived the infection showed just a transitory peak of body temperature, with viremias peaking by day 7 pi (with titers of 10^6^–10^7^ HAD_50_/mL). They kept similar viremia until day 28 pi. The other two remaining animals showed almost undetectable viremia values until day 28 pi (Figure 5). Therefore, ASFV-G-∆A104R, along with showing an attenuated phenotype, produced in the inoculated animals very heterogeneous patterns of viremia that do not correlate with the presence of clinical signs of ASF. This is not unique to ASFV-G-∆A104R, since it has also been seen with other attenuated strains we have produced in our laboratory [8,10,11,21].

**Figure 6:**
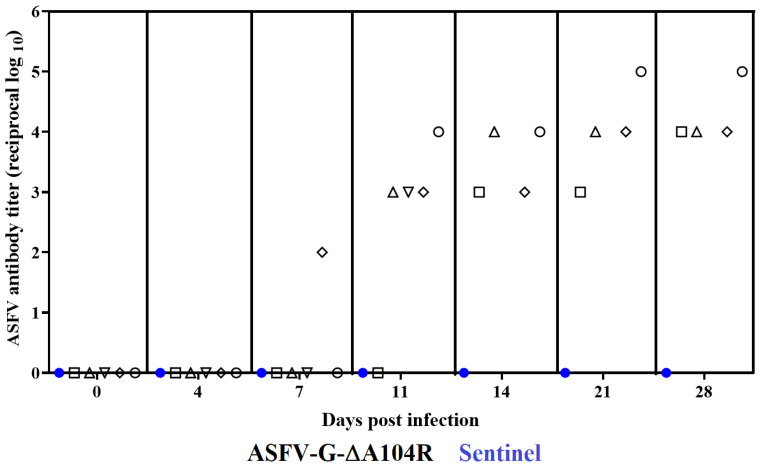
Anti-ASFV antibody titers detected by ELISA in pigs IM inoculated with 10^2^ HAD_50_ of ASFV-G-∆A104R. Each point represents values from individual animals.

Usually, animals surviving an ASFV infection develop a systemic virus-specific antibody response. Analysis of circulating ASFV specific antibodies were performed using an in house developed ELISA [33]. While an animal euthanized by day 9 pi is not able to develop an antibody response, ASFV specific circulating antibodies start to be detected by day 7 pi. The antibody response is clearly detected in almost all animals by day 11 pi and is firmly established by day 14 pi with all four animals reaching day 28 pi showing high antibody titers (Figure 6).

### 3.5. Evaluation of the Protective Effect of the Infection with ASFV-G-∆A104R

Animals surviving an infection with an ASFV strain generally became protected from the infection with the virulent homologous or parental virus [9,10,12,22,34,35,36,37]. Therefore, it was important to assess if animals infected with ASFV-G-∆A104R were protected against the infection with the parental virus, the highly virulent ASFV-G. Twenty-eight days after ASFV-G-∆A104R infection, the four surviving pigs were challenged by IM with 10^2^ HAD_50_ of ASFV-G. A group (*n* = 4) of naïve animals was used as a control group and challenged under the same conditions.

**Figure 7:**
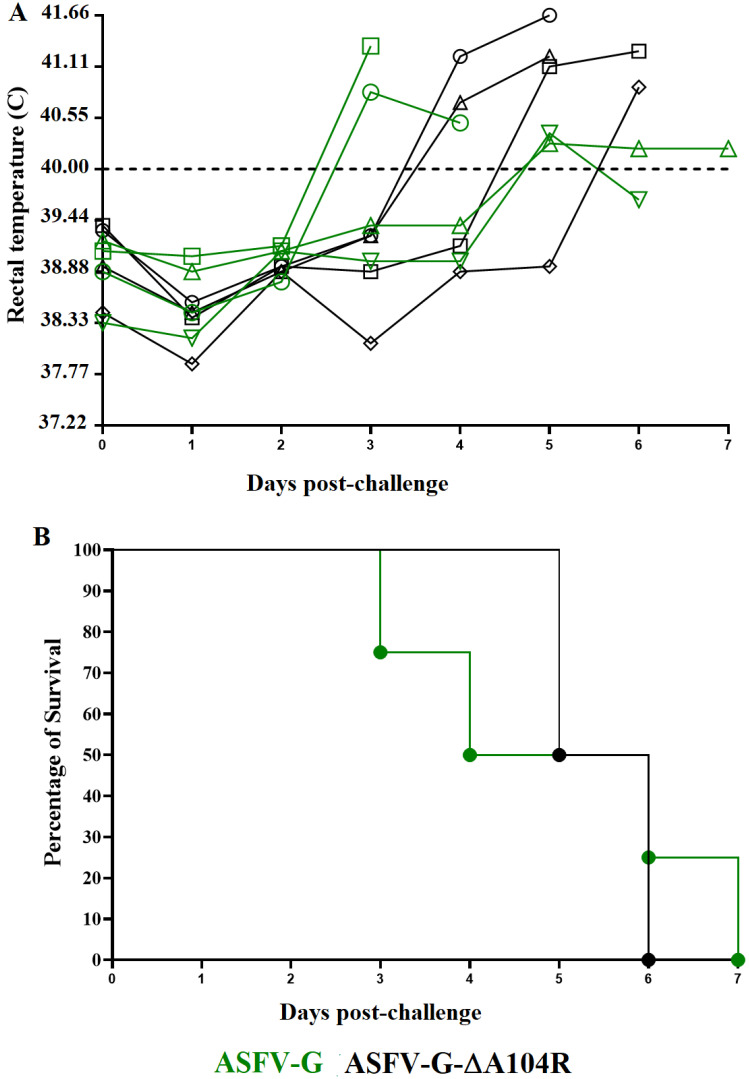
Evolution of body temperature (**A**) and mortality (**B**) in animals (4 animals/group) IM infected with 10^2^ HAD_50_ of ASFV-G-∆A104R and challenge 28 days later with 10^2^ HAD_50_ of parental ASFV-G.

Animals in the control group presented clinical signs of ASF by days 3 to 5 post challenge (dpc), which quickly evolved to a severe form of the disease terminating in all animals being euthanized by day 3 and 7 pc (Table 2, Figure 7). Similarly, animals previously infected with ASFV-G-∆A104R developed a clinical disease almost indistinguishable from animals in the control group. Therefore, infection with ASFV-G-∆A104R is unable to induce protection against a lethal form of the disease when challenged with the highly virulent ASFV-G.

Viremia titers in these animals followed the evolution of clinical signs presented by the animals challenged with ASFV-G. In the control group, titers were high (ranging between 10^7.5^–10^8.3^ HAD_50_/mL) at day 4 pi, remaining high until animals were euthanized due to the severity of the clinical disease (Figure 5). After challenge, viremia titers of in the ASFV-G-∆A104R infected animals ranged between 10^4.6^–10^6^ HAD_50_/mL) and had increased to approximately 10^7^ HAD_50_/mL when animals were euthanized.

Results presented here clearly indicate that the deletion of A104R, although it partially affects the ability of ASFV to replicate in the natural target cell, the macrophages, is not an essential viral gene, as was previously suggested [32]. Differences between those previous results and those presented here may be due to using viruses with important genomic differences. While here we used a highly virulent natural field isolate, those authors worked with a virus adapted to grow in Vero cells [32], which harbors the deletion of large areas on both sides of the virus genome [20]. Perhaps the lack of several virus genes may account for the lack of the potential vicarious complementary functions of the A104R gene.

It is also clear that the A104R gene is involved in the process of virus virulence in domestic pigs. All animals inoculated with ASFV-G-∆A104R survived the infection, with the exception of one, which presented a protracted fatal disease compared to that of the animals inoculated with the parental virus. This would indicate that ASFV-G-∆A104R has some level of residual virulence. This observation is not unusual since we have seen similar heterogeneous behavior in animals inoculated with recombinant viruses having residual virulence, and in naturally attenuated field isolates where a percentage of inoculated animals can have a protracted but fatal disease. The attenuation of ASFV virulence by deletion of a single gene is not a very common feature. Actually, only nine individual genes have been reported to produce attenuation of ASFV virulence: I177L [10,38], 9Gl [12,16], CD2 [14,15], A137 [11], I226R [39], I267L [40], NL [41], UK [42], and DP148R [43], and only five of these genes in the ASFV-G isolate or its derivative viruses: I177L [10,38], 9Gl [12], A137 [11], I226R [39], and I267L [40]. Although the deletion of the A104R gene does not completely attenuate ASFV-G, it could potentially be used to increase the safety of already attenuated strains. The deletion of genes such as CD2 [8], UK [44], or NL [44], which do not produce full attenuation when individually deleted, however, has been shown to enhance the attenuation profile when combined with other genes deletion [22,44,45].

It is interesting to mention the apparent absence of virus shedding in the animals inoculated with ASFV-G-∆A104R, even from the pig developing full lethal disease and need to be euthanized on day 9 pi. The naïve sentinel animal cohabiting for 28 days with the inoculated ones did not show any clinical sign of disease. It also did not show detectable virus in blood nor developed virus-specific antibodies.

Another interesting issue is that although all animals surviving ASFV-G-∆A104R infection developed high ASFV specific circulating antibody titers, none of them were protected against the experimental infection with the parental virulent strain ASFV-G. This is a rather unusual outcome since, in our experience, animals showing such high antibody titers after infection with different recombinant attenuated virus strains developed solid protection, at least, against disease presentation [9,10,11,12,13,35]. At this point, we do not understand the basis of this particular differential behavior between ASFV-G-∆A104R and other attenuated strains.

In summary, we determined that A104R is a non-essential gene, since its deletion from the ASFV-G genome does not significantly alter virus replication in swine macrophage cultures and it is one of the few individual genes whose deletion causes a decrease in virus virulence of the highly virulent ASFV-G strain. Identification of ASFV genes involved in disease production is a critical first step in the rational development of recombinant viruses that can be potentially used as live attenuated vaccine candidates.

## Figures and Tables

**Table 1:** Swine survival and fever response following infection with ASFV-G-∆A104R and parental ASFV-G.

Virus (10^2^ HAD_50_)	No. of Survivors/Total	Mean Time to Death (±SD)	Fever		
			No. of Days to Onset (±SD)	Duration No. of Days (±SD)	Maximum Daily Temp, °C (±SD)
ASFV-G	0/5	6.25 (0.55)	4.4 (0.84)	2 (1.1)	40.53 (0.55)
ASFV-G-ΔA104R	4/5	8 *	5	3	40.14 (1.51)

(*) Data are calculated based on the only animal euthanized in this group.

**Table 2:** Swine survival and fever response following infection with ASFV-G-∆A104R and challenged 28 days later with ASFV-G.

Virus (10^2^ HAD_50_)	No. of Survivors/Total	Mean Time to Death (±SD)	Fever		
			No. of Days to Onset (±SD)	Duration No. of Days (±SD)	Maximum Daily Temp, °C (±SD)
Mock	0/4	5 (1.83)	4 (1.15)	1 (0.82)	40.06 (0.89)
ASFV-G-ΔA104R	0/4	5.5 (1.5)	4.75 (0.95)	0.75 (0.5)	40.75 (0.27)

## References

[1] Gonzales W., Moreno C., Duran U., Henao N., Bencosme M., Lora P., Reyes R., Nunez R., De Gracia A., Perez A.M. (2021). African swine fever in the Dominican Republic. Transbound. Emerg. Dis..

[2] Tulman E.R., Delhon G.A., Ku B.K., Rock D.L., Etten V. (2009). African Swine Fever Virus. Lesser Known Large dsDNA Viruses. Current Topics in Microbiology and Immunology.

[3] Ramirez-Medina E., Vuono E.A., Pruitt S., Rai A., Espinoza N., Velazquez-Salinas L., Gladue D.P., Borca M.V. (2021). Evaluation of an ASFV RNA Helicase Gene A859L for Virus Replication and Swine Virulence. Viruses.

[4] Vuono E.A., Ramirez-Medina E., Pruitt S., Rai A., Espinoza N., Velazquez-Salinas L., Gladue D.P., Borca M.V. (2021). Evaluation of the Function of the ASFV KP177R Gene, Encoding for Structural Protein p22, in the Process of Virus Replication and in Swine Virulence. Viruses.

[5] Ramirez-Medina E., Vuono E., Pruitt S., Rai A., Silva E., Espinoza N., Zhu J., Velazquez-Salinas L., Borca M.V., Gladue D.P. (2021). Development and In Vivo Evaluation of a MGF110-1L Deletion Mutant in African Swine Fever Strain Georgia. Viruses.

[6] Ramirez-Medina E., Vuono E., Pruitt S., Rai A., Silva E., Zhu J., Velazquez-Salinas L., Gladue D.P., Borca M.V. (2020). X69R Is a Non-Essential Gene That, When Deleted from African Swine Fever, Does Not Affect Virulence in Swine. Viruses.

[7] Ramirez-Medina E., Vuono E.A., Rai A., Pruitt S., Silva E., Velazquez-Salinas L., Zhu J., Gladue D.P., Borca M.V. (2020). Evaluation in Swine of a Recombinant African Swine Fever Virus Lacking the MGF-360-1L Gene. Viruses.

[8] Borca M.V., O’Donnell V., Holinka L.G., Risatti G.R., Ramirez-Medina E., Vuono E.A., Shi J., Pruitt S., Rai A., Silva E. (2020). Deletion of CD2-like gene from the genome of African swine fever virus strain Georgia does not attenuate virulence in swine. Sci. Rep..

[9] Borca M.V., Ramirez-Medina E., Silva E., Vuono E., Rai A., Pruitt S., Espinoza N., Velazquez-Salinas L., Gay C.G., Gladue D.P. (2021). ASFV-G-I177L as an Effective Oral Nasal Vaccine against the Eurasia Strain of Africa Swine Fever. Viruses.

[10] Borca M.V., Ramirez-Medina E., Silva E., Vuono E., Rai A., Pruitt S., Holinka L.G., Velazquez-Salinas L., Zhu J., Gladue D.P. (2020). Development of a Highly Effective African Swine Fever Virus Vaccine by Deletion of the I177L Gene Results in Sterile Immunity against the Current Epidemic Eurasia Strain. J. Virol..

[11] Gladue D.P., Ramirez-Medina E., Vuono E., Silva E., Rai A., Pruitt S., Espinoza N., Velazquez-Salinas L., Borca M.V. (2021). Deletion of the A137R Gene from the Pandemic Strain of African Swine Fever Virus Attenuates the Strain and Offers Protection against the Virulent Pandemic Virus. J. Virol..

[12] O’Donnell V., Holinka L.G., Krug P.W., Gladue D.P., Carlson J., Sanford B., Alfano M., Kramer E., Lu Z., Arzt J. (2015). African swine fever virus Georgia 2007 with a deletion of virulence-associated gene 9GL (B119L), when administered at low doses, leads to virus attenuation in swine and induces an effective protection against homologous challenge. J. Virol..

[13] Ramirez-Medina E., Vuono E., Rai A., Pruitt S., Espinoza N., Velazquez-Salinas L., Pina-Pedrero S., Zhu J., Rodriguez F., Borca M.V. (2022). Deletion of E184L, a Putative DIVA Target from the Pandemic Strain of African Swine Fever Virus, Produces a Reduction in Virulence and Protection against Virulent Challenge. J. Virol..

[14] Monteagudo P.L., Lacasta A., Lopez E., Bosch L., Collado J., Pina-Pedrero S., Correa-Fiz F., Accensi F., Navas M.J., Vidal E. (2017). BA71DeltaCD2: A New Recombinant Live Attenuated African Swine Fever Virus with Cross-Protective Capabilities. J. Virol..

[15] Borca M.V., Carrillo C., Zsak L., Laegreid W.W., Kutish G.F., Neilan J.G., Burrage T.G., Rock D.L. (1998). Deletion of a CD2-like gene, 8-DR, from African swine fever virus affects viral infection in domestic swine. J. Virol..

[16] Lewis T., Zsak L., Burrage T.G., Lu Z., Kutish G.F., Neilan J.G., Rock D.L. (2000). An African swine fever virus ERV1-ALR homologue, 9GL, affects virion maturation and viral growth in macrophages and viral virulence in swine. J. Virol..

[17] Borca M.V., Irusta P.M., Kutish G.F., Carillo C., Afonso C.L., Burrage A.T., Neilan J.G., Rock D.L. (1996). A structural DNA binding protein of African swine fever virus with similarity to bacterial histone-like proteins. Arch. Virol..

[18] Frouco G., Freitas F.B., Coelho J., Leitao A., Martins C., Ferreira F. (2017). DNA-Binding Properties of African Swine Fever Virus pA104R, a Histone-Like Protein Involved in Viral Replication and Transcription. J. Virol..

[19] Borca M.V., Berggren K.A., Ramirez-Medina E., Vuono E.A., Gladue D.P. (2018). CRISPR/Cas Gene Editing of a Large DNA Virus: African Swine Fever Virus. Bio-Protocol.

[20] Krug P.W., Holinka L.G., O’Donnell V., Reese B., Sanford B., Fernandez-Sainz I., Gladue D.P., Arzt J., Rodriguez L., Risatti G.R. (2015). The progressive adaptation of a Georgian isolate of African swine fever virus to Vero cells leads to a gradual attenuation of virulence in swine corresponding to major modifications of the viral genome. J. Virol..

[21] Reed L.J., Muench H. (1938). A simple method of estimating fifty percent endpoints. Am. J. Hyg..

[22] O’Donnell V., Risatti G.R., Holinka L.G., Krug P.W., Carlson J., Velazquez-Salinas L., Azzinaro P.A., Gladue D.P., Borca M.V. (2017). Simultaneous deletion of the 9GL and UK genes from the African swine fever virus Georgia 2007 isolate offers increased safety and protection against homologous challenge. J. Virol..

[23] Borca M.V., O’Donnell V., Holinka L.G., Sanford B., Azzinaro P.A., Risatti G.R., Gladue D.P. (2017). Development of a fluorescent ASFV strain that retains the ability to cause disease in swine. Sci. Rep..

[24] Kumar S., Stecher G., Li M., Knyaz C., Tamura K. (2018). MEGA X: Molecular Evolutionary Genetics Analysis across Computing Platforms. Mol. Biol. Evol..

[25] Neilan J.G., Lu Z., Kutish G.F., Sussman M.D., Roberts P.C., Yozawa T., Rock D.L. (1993). An African swine fever virus gene with similarity to bacterial DNA binding proteins, bacterial integration host factors, and the Bacillus phage SPO1 transcription factor, TF1. Nucleic Acids Res..

[26] Kosakovsky Pond S.L., Frost S.D. (2005). Not so different after all: A comparison of methods for detecting amino acid sites under selection. Mol. Biol. Evol..

[27] Weaver S., Shank S.D., Spielman S.J., Li M., Muse S.V., Kosakovsky Pond S.L. (2018). Datamonkey 2.0: A Modern Web Application for Characterizing Selective and Other Evolutionary Processes. Mol. Biol. Evol..

[28] Kosakovsky Pond S.L., Posada D., Gravenor M.B., Woelk C.H., Frost S.D. (2006). GARD: A genetic algorithm for recombination detection. Bioinformatics.

[29] Urbano A.C., Ferreira F. (2020). Role of the DNA-Binding Protein pA104R in ASFV Genome Packaging and as a Novel Target for Vaccine and Drug Development. Vaccines.

[30] Murrell B., Wertheim J.O., Moola S., Weighill T., Scheffler K., Kosakovsky Pond S.L. (2012). Detecting individual sites subject to episodic diversifying selection. PLoS Genet..

[31] Alejo A., Matamoros T., Guerra M., Andres G. (2018). A Proteomic Atlas of the African Swine Fever Virus Particle. J. Virol..

[32] Freitas F.B., Simoes M., Frouco G., Martins C., Ferreira F. (2019). Towards the Generation of an ASFV-pA104R DISC Mutant and a Complementary Cell Line-A Potential Methodology for the Production of a Vaccine Candidate. Vaccines.

[33] Carlson J., O’Donnell V., Alfano M., Velazquez Salinas L., Holinka L.G., Krug P.W., Gladue D.P., Higgs S., Borca M.V. (2016). Association of the Host Immune Response with Protection Using a Live Attenuated African Swine Fever Virus Model. Viruses.

[34] Zhang J., Zhang Y., Chen T., Yang J., Yue H., Wang L., Zhou X., Qi Y., Han X., Ke J. (2021). Deletion of the L7L-L11L Genes Attenuates ASFV and Induces Protection against Homologous Challenge. Viruses.

[35] O’Donnell V., Holinka L.G., Gladue D.P., Sanford B., Krug P.W., Lu X., Arzt J., Reese B., Carrillo C., Risatti G.R. (2015). African Swine Fever Virus Georgia Isolate Harboring Deletions of MGF360 and MGF505 Genes Is Attenuated in Swine and Confers Protection against Challenge with Virulent Parental Virus. J. Virol..

[36] Li D., Liu Y., Qi X., Wen Y., Li P., Ma Z., Liu Y., Zheng H., Liu Z. (2021). African Swine Fever Virus MGF-110-9L-deficient Mutant Has Attenuated Virulence in Pigs. Virol. Sin..

[37] Li D., Yang W., Li L., Li P., Ma Z., Zhang J., Qi X., Ren J., Ru Y., Niu Q. (2021). African Swine Fever Virus MGF-505-7R Negatively Regulates cGAS-STING-Mediated Signaling Pathway. J. Immunol..

[38] Tran X.H., Le T.T.P., Nguyen Q.H., Do T.T., Nguyen V.D., Gay C.G., Borca M.V., Gladue D.P. (2021). African swine fever virus vaccine candidate ASFV-G-DeltaI177L efficiently protects European and native pig breeds against circulating Vietnamese field strain. Transbound. Emerg. Dis..

[39] Zhang Y., Ke J., Zhang J., Yang J., Yue H., Zhou X., Qi Y., Zhu R., Miao F., Li Q. (2021). African Swine Fever Virus Bearing an I226R Gene Deletion Elicits Robust Immunity in Pigs to African Swine Fever. J. Virol..

[40] Ran Y., Li D., Xiong M.G., Liu H.N., Feng T., Shi Z.W., Li Y.H., Wu H.N., Wang S.Y., Zheng H.X. (2022). African swine fever virus I267L acts as an important virulence factor by inhibiting RNA polymerase III-RIG-I-mediated innate immunity. PLoS Pathog..

[41] Zsak L., Lu Z., Kutish G.F., Neilan J.G., Rock D.L. (1996). An African swine fever virus virulence-associated gene NL-S with similarity to the herpes simplex virus ICP34.5 gene. J. Virol..

[42] Zsak L., Caler E., Lu Z., Kutish G.F., Neilan J.G., Rock D.L. (1998). A nonessential African swine fever virus gene UK is a significant virulence determinant in domestic swine. J. Virol..

[43] Reis A.L., Goatley L.C., Jabbar T., Sanchez-Cordon P.J., Netherton C.L., Chapman D.A.G., Dixon L.K. (2017). Deletion of the African Swine Fever Virus Gene DP148R Does Not Reduce Virus Replication in Culture but Reduces Virus Virulence in Pigs and Induces High Levels of Protection against Challenge. J. Virol..

[44] Ramirez-Medina E., Vuono E., O’Donnell V., Holinka L.G., Silva E., Rai A., Pruitt S., Carrillo C., Gladue D.P., Borca M.V. (2019). Differential Effect of the Deletion of African Swine Fever Virus Virulence-Associated Genes in the Induction of Attenuation of the Highly Virulent Georgia Strain. Viruses.

[45] Teklue T., Wang T., Luo Y., Hu R., Sun Y., Qiu H.J. (2020). Generation and Evaluation of an African Swine Fever Virus Mutant with Deletion of the CD2v and UK Genes. Vaccines.

